# Selection of essential medicines for the prevention and treatment of cardiovascular diseases in low and middle income countries

**DOI:** 10.1186/s12872-018-0858-5

**Published:** 2018-06-25

**Authors:** Y. T. Bazargani, M. Ugurlu, A. de Boer, H. G. M. Leufkens, A. K. Mantel-Teeuwisse

**Affiliations:** Division of Pharmacoepidemiology and Clinical Pharmacology, Utrecht Institute for Pharmaceutical Sciences, Utrecht University, PO Box 80082, 3508 TB Utrecht, The Netherlands

**Keywords:** Cardiovascular diseases, Low and middle income countries, Essential medicines lists, Access to medicines

## Abstract

**Background:**

The incidence and mortality of cardiovascular diseases (CVDs) in low and middle income countries (LMICs) have been increasing, while access to CVDs medicines is suboptimal. We assessed selection of essential medicines for the prevention and treatment of CVDs on national essential medicines lists (NEMLs) of LMICs and potential determinants for selection.

**Methods:**

Only operational NEMLs were considered eligible for this study. A selection of medicines listed under “cardiovascular medicines” or “blood products and plasma substitutes” in the NEMLs were included if they were present on international guidelines for the prevention and treatment of CVDs (hyperlipidemia, hypertension, platelet inhibition, ischemic stroke, stable ischemic heart disease, acute coronary syndromes, heart failure, atrial fibrillation, peripheral arterial disease and acute limb ischemia). The number and diversity of essential medicines selected for CVDs were studied. Moreover, determinants of selection of essential medicines for CVDs at a national level were explored. Data analysis was done using univariate linear regression and non-parametric tests.

**Results:**

All medicine groups listed by the international guidelines were selected by the majority of the 34 countries studied with the exception of adenosine diphosphate receptor inhibitors which appeared on less than half of the NEMLs studied (41% of countries). The total number of essential medicines for the prevention and treatment of cardiovascular diseases (median 24 (range 16–50)) differed significantly across income levels (median range: 19.5–25, *p* = 0.014) and across regions (median range: 20–32, *p* = 0.049). When recommendations of the international guidelines were considered, over 75% of the NEMLs contained essential medicines for the majority of CVDs.

**Conclusion:**

The main medicine classes for the management of CVDs were represented on NEMLs. Consequently, for the majority of CVDs, evidence-based guideline-recommended treatment is possible as far as selection of essential medicines is concerned. Selection will therefore not be the limiting step in access to medicines for cardiovascular diseases.

**Electronic supplementary material:**

The online version of this article (10.1186/s12872-018-0858-5) contains supplementary material, which is available to authorized users.

## Background

Cardiovascular diseases (CVDs) are the most common cause of death worldwide with more than 17 million deaths annually [1]. Global estimates show that CVDs such as ischemic heart disease and cerebrovascular disease will still be the primary cause of death by 2030 and will be associated with productivity loss and catastrophic healthcare costs [2, 3].

Ongoing changes in low and middle income countries (LMICs), accelerated by urbanization and socio-economic development, have increased the exposure to health related risks such as tobacco smoking, unhealthy diet and reduced physical activity [4]. Together with ageing of the population these changes have led to an increase in the incidence of non-communicable diseases including CVDs in these countries [1, 4]. Appropriate preventive measures should be taken to slow down this detrimental developments and treatment of these diseases should be prioritized. This notion has been accentuated in various international meetings and governments have made a variety of commitments in this direction [5, 6]. Evidence indicates that more than 80% of global cardiovascular deaths occur in LMICs which is (partly) due to the lack of access to healthcare including skilled human resources, equipped facilities and medicines [7, 8]. Medicines are more available for treatment of infectious disease as opposed to CVDs or other non-communicable diseases [9]. In order to change this inequality, essential medicines could be instrumental. The WHO has compiled and revises a list of medicines which is considered essential to meet global health needs, the so-called WHO essential medicines list. It is recommended by the WHO that countries make use of this list as a guide to prepare their own national essential medicines lists (NEMLs). A NEML is supposed to respond to the health care priorities of each individual country as determined by the national burden of disease and national health care priorities. It is shown that essential medicines are more available than other medicines across LMICs, hence NEMLs play indeed a role in supply of medicines (at least) in the public sector. A NEML often constitutes a basis for district level medicines lists and hospital formularies [10, 11]. Therefore, a preliminary step in guaranteeing equitable access to medicines in LMICs, is adopting a NEML with a rational and balanced approach in selection of essential medicines.

This study will assess selection of essential medicines for the prevention and treatment of a selection of CVDs on NEMLs of LMICs. Potential determinants for this selection, namely income level and geographic region of countries, national burden of CVDs and update of NEMLs on selection will be studied. Additionally, the extent to which different CVDs can be treated according to the guidelines by the selected essential medicines will be explored.

## Methods

### Selection of countries and medicines

The latest available updates of NEMLs at data collection (August 2015), were obtained from the “WHO database of essential medicine lists, formularies and treatment guidelines” [12]. Of these, only NEMLs of countries in which the NEML is routinely used for procurement and/or reimbursement purposes -according to the WHO Pharmaceutical Sector Country Profiles survey- were included [13] (Additional file 1). The Country Profiles survey repository data gets updated once a newer revision of the survey report becomes available for each country.

Medicines listed on the NEMLs were included if they were categorized under “cardiovascular medicines” or “blood products and plasma substitutes” or similar naming and classification in other languages and if they were present in international guidelines for the prevention and treatment of a selection of CVDs (see the therapeutic guidelines section below).

### Data sources for determinants of selection

Data on income levels and geographic regions were obtained from the World Bank and the WHO, respectively [14, 15]. The burden of CVDs was obtained for each country from the Institute for Health Metrics and Evaluation [16]. The total burden of CVDs was expressed as the Years of Life Lost (YLL) and Years Lived with Disabilities (YLD) with regard to mortality and morbidity of CVDs, respectively.

### Therapeutic guidelines

In order to assess whether the medicines listed on the NEMLs were sufficient enough to provide pharmaceutical treatment for different CVDs, the latest update of international treatment guidelines for CVD management - found following a thorough search in PubMed and other available data sources (e.g. WHO guidelines, cardiovascular associations) were consulted [17–48]. Unless a guideline was revised and updated, we assumed that the latest revision of the guideline is still valid. The recommendations of the guidelines were classified into two sections, namely recommendations for primary and secondary prevention of CVDs and recommendations for treatment of (acute) cardiovascular diseases. For prevention, medicines indicated to treat hyperlipidemia and hypertension and platelet inhibitors were considered. Medicines to treat diabetes, obesity and smoking were not included. For the cardiovascular diseases medicines necessary to treat ischemic stroke (including transient ischemic attacks (TIA) and cerebrovascular accidents (CVA)), stable ischemic heart disease (including stable angina pectoris (AP)), acute coronary syndromes (including unstable AP, ST segment elevation myocardial infarction (STEMI) and non-STEMI), heart failure, atrial fibrillation, peripheral arterial disease (intermittent claudication) and acute limb ischemia were studied (Table 1).

**Table 1:** ᅟ

Primary and secondary prevention of cardiovascular diseases	Hypertension treatment	thiazide diuretics, selective β1-blockers, dihydropyridine calcium channel blockers, renin-angiotensin-aldosterone system (RAAS) inhibitors including angiotensin-converting enzyme (ACE) inhibitors or angiotensin receptor blockers (ARBs)
	Dyslipidaemia treatment	statins
	Platelet inhibition	acetylsalicylic acid, adenosine diphosphate (ADP) receptor inhibitors
Treatment of acute cardiovascular events	Ischemic stroke	thrombolytic agents including streptokinase and urokinase as well as recombinant tissue plasminogen activators (rt-PAs), platelet inhibitors (acetyl salicylic acid or ADP-receptor blockers)
	Stable ischemic heart disease	Angina attack treatment: nitroglycerin Prophylaxis angina pectoris attacks: selective β1-blockers, long-acting nitrates, non-dihydropyridine calcium channel blockers
	Acute coronary syndrome	nitrates, morphine, selective β1-blockers, acetyl salicylic acid, ADP-receptor blockers, heparin-like medicines (including unfractionated heparin (UFH) and low molecular weight heparins (LMWH)), thrombolytic agents Management of further complications of ACS: Ventricular fibrillation: lidocaine, Bradycardia: atropine, Cardiogenic shock: dopamine
	Heart failure	loop diuretics, RAAS inhibitors, selective β1-blockers, aldosteron antagonists, digitalis glycosides, dopamine or dobutamine or milrinone
	Atrial fibrillation	oral anticoagulants including vitamin K antagonists or direct oral anticoagulants (DOAC) Frequency control**:** selective β1-blockers, non-dihydropyridine calcium channel blockers, digitalis glycosides (in case of atrial fibrillation plus heart failure) Rhythm control: pharmacological cardioversion: flecainide, dofetilide, propafenone, ibutilide, amiodarone maintaining sinus rhythm: amiodarone, dofetilide, dronedarone, flecainide, propafenone, sotalol
	Peripheral arterial disease (Intermittent claudication)	cilostazol or naftidrofuryl (for relief of ischemic complaints)
	Acute limb ischemia	heparin, thrombolytic agents, platelet inhibitors

### Data analysis

We hypothesized that number of essential medicines selected for the prevention and treatment of CVDs on an NEML varied across different country income levels (with upper middle income countries having more essential medicines), geographic regions, and burden of disease (with countries with higher CVD burden having more essential medicines). The numbers of essential medicines for CVDs according to our inclusion criteria (in total and within each medicine group included in Table 1) were retrieved from each NEML. Results were then stratified by different geographic regions and income levels. Because the WHO published a revision of the model list of essential medicines in 2009, the number of listed essential medicines was compared between NEMLs released from 2009 onwards and NEMLs released prior to 2009. Non-parametric tests (Mann-Whitney U and Kruskal Wallis tests) were used to test for statistical significance for differences across different clusters of countries. To examine the association between the continuous variables (burden of disease) and the number of essential cardiovascular medicines, univariate linear regression analysis was used. As for the guidelines, the number of countries which selected at least one essential medicine from those medicine classes recommended by the international guidelines for prevention and treatment of CVDs was studied and related statistics was collated.

## Results

### Selection of essential medicines

A total of 34 countries across 6 different regions were included in the study (see Additional file 1). The overall median number of essential medicines selected for the prevention and treatment of cardiovascular diseases on NEMLs was 24 (range 16–50) (Table 2). The majority of the countries had selected one medicine from each of the main medicine groups indicated for the cardiovascular diseases included in this study with the exception of dihydropyridine calcium channel blockers (CCBs) and renin-angiotensin-aldosterone system (RAAS) inhibitors (with a median of 2 medicines selected for each group) and adenosine diphosphate (ADP) receptor inhibitors with a median of 0 selected medicines.

**Table 2:** ᅟ

	Total number of medicines for CVDs	Thiazide diuretics	Selective β1 blockers	Dihydropyridine CCBs	RAAS inhibitors^##^	Statins	ADP receptor inhibitors^##^	Thrombolytic agents	Long-acting nitrates	Non-dihydropyridine CCBs^##^	Heparin-like medicines	Loop diuretics	Aldosterone antagonists	Digitalis glycosides	Oral anticoagulants	Pharmacological cardioversion	Medicines for maintaining sinus rhythm
Overall	24 (16-50)	1 (0-2)	1 (1-5)	2 (1-4)	2(1-11)	1(0-4)	0(0-2)	1(0-2)	1(0-2)	1(0-3)	1(1-4)	1(1-2)	1(1-1)	1(1-2)	1(0-2)	1(0-3)	1(0-4)
Low income(*n* = 6)	19.5(16-24)	1(1-2)	1(1-2)	2(1-3)	2(1-2)	0.5(0-2)	0(0-0)	0.5(0-2)	1(0-1)	1(0-1)	1(1-2)	1(1-2)	1(1-1)	1(1-2)	1(0-2)	0.5(0-1)	0.5(0-1)
Lower middle income (*n* = 11)	20(16-44)	1(1-2)	1(1-3)	2(1-3)	2(1-6)	1(0-4)	0(0-2)	1(0-2)	1(1-2)	1(1-2)	1(1-4)	1(1-2)	1(1-1)	1(1-1)	1(1-2)	1(0-3)	1(0-4)
Upper middle income (*n* = 17)	25(17-50)	1(0-2)	2(1-5)	2(1-4)	2(1-11)	1(0-4)	1(0-2)	1(0-2)	1(1-2)	2(0-3)	2(1-3)	1(1-2)	1(1-1)	1(1-2)	1(0-2)	1(0-3)	1(0-3)
*p*-value*	0.014	0.258	0.167	0.824	0.080	0.194	0.087	0.547	0.013	0.001	0.542	0.901	1.000	0.133	0.701	0.052	0.043
AMRO^₸^(*n* = 9)	32(20-39)	1(0-2)	2(1-3)	2(1-4)	3(2-7)	2(1-4)	1(0-1)	1(0-2)	2(1-2)	2(1-3)	2(1-4)	1(1-1)	1(1-1)	1(1-2)	1(1-2)	1(1-3)	1(1-3)
AFRO(*n* = 9)	20(16-44)	1(0-2)	1(1-3)	2(1-3)	2(1-6)	0(0-4)	0(0-2)	1(0-2)	1(0-1)	1(0-2)	1(1-2)	1(1-2)	1(1-1)	1(1-2)	1(1-2)	1(0-3)	1(0-4)
WPRO(*n* = 7)	21(16-26)	1(1-2)	1(1-3)	1(1-2)	2(2-2)	1(0-1)	0(0-1)	1(0-1)	1(1-2)	1(0-3)	1(1-2)	1(1-1)	1(1-1)	1(1-2)	1(0-1)	1(0-2)	1(0-2)
SEARO(*n* = 5)	27(20-35)	1(1-2)	2(1-3)	2(1-2)	2(1-3)	1(1-1)	1(1-2)	1.5(1-2)	1.5(1-2)	1.5(1-2)	2(2-3)	1(1-1)	1(1-1)	1(1-1)	1(1-1)	1(1-3)	1(1-3)
EMRO(*n* = 4)	22(18-50)	1(1-1)	2(1-5)	2.5(1-4)	3(1-11)	1(1-4)	0(0-2)	1(0-2)	1(1-1)	1(1-2)	2(1-3)	1(1-2)	1(1-1)	1(1-1)	1(0-1)	0.5(0-3)	0.5(0-3)
*p*-value**	0.049	0.833	0.204	0.205	0.013	0.033	0.059	0.255	0.011	0.379	0.029	0.138	1.000	0.746	0.118	0.241	0.192
WHO ^#^	21	1^$^	1^$^	1^$^	1^$^	1^$^	1	1	1^$^	1	2^$^	1	1	1	1^$^	1	1

### Factors associated with selection of essential medicines

The number of essential medicines selected for the prevention and treatment of cardiovascular diseases on the NEMLs differed significantly across income levels (median range: 19.5–25, *p* = 0.014) and across regions (median range: 20–32, *p* = 0.049) (Table 2). In addition, the number of selected medicines differed significantly across income levels for long-acting nitrates, medicines for maintaining sinus rhythm and non-dihydropyridine CCBs and across regions for statins (with a median of 0 selected medicines in Africa), long-acting nitrates, heparin-like medicines and RAAS inhibitors. Despite the fact that the number of medicines selected from the group of RAAS inhibitors did not differ significantly across income levels (*p* = 0.080), none of the low income countries selected medicines from the subgroup of angiotensin receptor blockers (ARBs). In addition, selection of evidence based beta-blockers for heart failure (see further details below) differed significantly across income levels (*p* = 0.005), being less frequently selected by low income countries, and across regions (*p* = 0.003). Selection of medicines which were studied as single medicine from their class such as acetylsalicylic acid, nitroglycerin, lidocaine, dopamine, dobutamine, milrinone did not differ significantly across income levels and regions.

The median number of essential medicines on the NEMLs released from 2009 onwards did not differ significantly from NEMLs prior to 2009. No significant associations were found between the numbers of essential medicines for the prevention and treatment of cardiovascular diseases and burden of CVDs for both YLD and YLL.

### Management of different CVDs according to the guidelines

#### Primary and secondary prevention of cardiovascular diseases

**Fig. 1:**
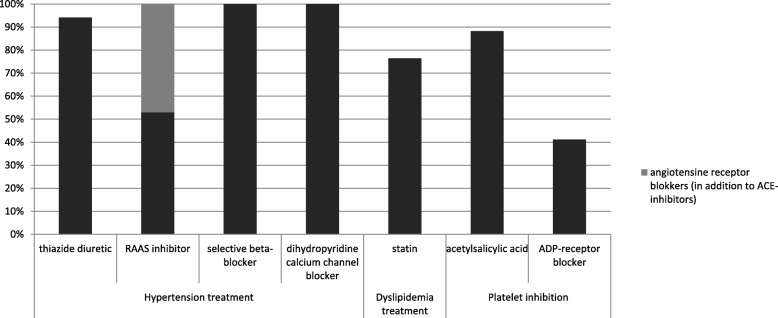
Percentage of 34 countries with essential medicines for primary and secondary prevention of cardiovascular diseases. RAAS inhibitors: renin angiotensin aldosterone system inhibitors, from which angiotensin-converting enzyme (ACE inhibitors) and angiotensin receptor blockers (ARBs) are included. Selective beta-blocker here refers to β1-selective agents, including: metoprolol, bisoprolol, acebutolol, atenolol, betaxolol, celiprolol, esmolol, nebivolol. ADP- receptor blockers: adenosine diphosphate receptor inhibitors (especially P2Y12 receptor inhibitors) include medicines such as clopidogrel, prasugrel, ticagrelor

Hypertension treatment including thiazide diuretics (e.g. hydrochlorothiazide), renin-angiotensin-aldosterone system (RAAS) inhibitors (e.g. enalapril or valsartan), selective beta-blockers (e.g. metoprolol) and dihydropyridine calcium channel blocker (e.g. amlodipine) was selected by 32 countries (94%) (Fig 1). From the RAAS inhibitors, angiotensin-converting enzyme (ACE) inhibitors were included in all NEMLs whereas angiotensin receptor blockers (ARBs) were selected by only 16 countries (47%). Statins to treat dyslipidaemia were selected by 26 countries (76%). Other lipid lowering agents did not appear in the NEMLs that did not include statins. Platelet inhibition therapy by acetylsalicylic acid was selected in 30 countries (88%). ADP-receptor blockers (e.g. clopidogrel) were only found in 14 countries (41%).

#### Treatment of acute cardiovascular events

##### Ischemic stroke

**Fig. 2:**
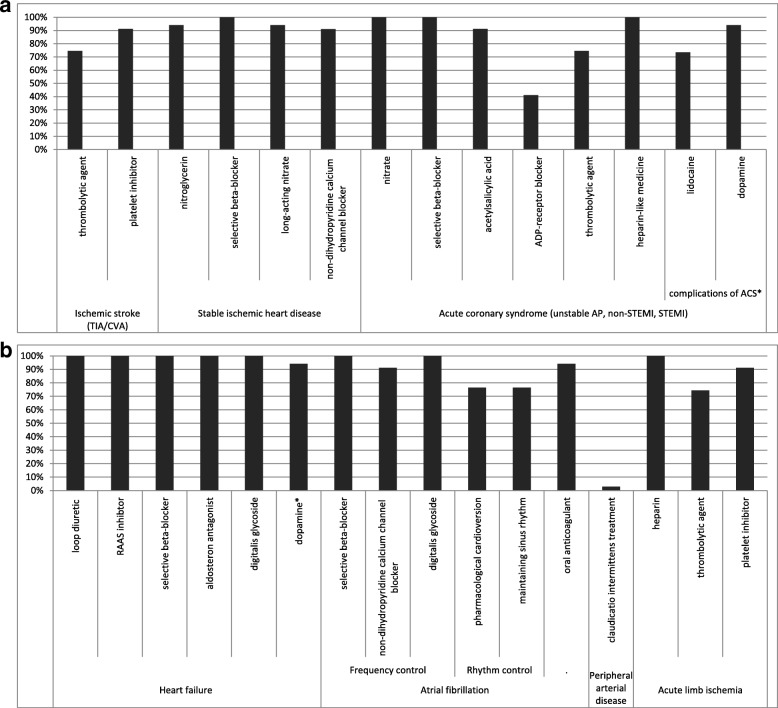
a- Percentage of 34 countries with essential medicines selected for treatment of acute cardiovascular events (ischemic stroke, stable ischemic heart disease, acute coronary syndrome). Thrombolytic agents includes streptokinase and urokinase as well as recombinant tissue plasminogen activators (rt-PAs) such as alteplase, reteplase, and tenecteplase. Platelet inhibitors include either acetylsalicylic acid or ADP-receptor blockers (e.g. clopidogrel). Selective beta-blocker here refers to β1-selective agents, including: metoprolol, bisoprolol, acebutolol, atenolol, betaxolol, celiprolol, esmolol, nebivolol. Heparin-like medicines includes unfractionated heparin (UFH) as well as low molecular weight heparins (LMWH) e.g. enoxaparin. Ischemic stroke includes both transient ischemic attacks (TIA) and cerebrovascular accidents (CVA). Acute coronary syndrome (ACS) refers to unstable angina pectoris (AP), ST segment elevation myocardial infarction (STEMI) and non-STEMI. Treatment of complications of ACS also requires atropine, which was out of the scope of this study. b - Percentage of 34 countries with essential medicines selected for treatment of acute cardiovascular events (heart failure, peripheral arterial disease, acute limb ischemia). RAAS inhibitors: Renin-angiotensin-aldosterone system inhibitors, from which angiotensin-converting enzyme (ACE) inhibitors and angiotensin receptor blockers (ARBs) are included in the table. Selective beta-blocker here refers to β1-selective agents, including: metoprolol, bisoprolol, acebutolol, atenolol, betaxolol, celiprolol, esmolol, nebivolol. Dopamine* includes dopamine, dobuamine, milrinone. Medicines for pharmacological cardioversion are flecainide, dofetilide, propafenone, ibutilide, amiodarone. Medicines for maintaining sinus rhythm are amiodarone, dofetilide, dronedarone, flecainide, propafenone, sotalol. Oral anticoagulants include both vitamin K antagonists (e.g. warfarin) as well as direct oral anticoagulants (DOAC; dabigatran, rivaroxaban, apixaban). Medicines for management of claudicatio intermittens include cilostazol or naftidrofuryl. Thrombolytic agents includes streptokinase and urokinase as well as recombinant tissue plasminogen activators (rt-PAs) such as alteplase, reteplase, and tenecteplase. Platelet inhibitors include either acetylsalicylic acid or ADP-receptor blockers (e.g. clopidogrel)

Intravenous thrombolytic (fibrinolytic) therapy including streptokinase, urokinase or recombinant tissue plasminogen activator (rt-PA) was selected in 25 countries (74%, Fig. 2a). Platelet inhibitors (acetyl salicylic acid or ADP-receptor blockers) were selected in 31 countries (91%).

##### Stable ischemic heart disease

Both treatment of angina attacks (short-acting nitroglycerin) and prophylactic therapy of angina pectoris (selective beta blockers, long-acting nitrates, non-dihydropyridine calcium channel blockers) were selected in over 90% of the countries. It has been recommended that at least two of the classes of these prophylactic therapies should be available in each country [23, 43, 45], which occurred in 32 countries (94%). The preferred calcium channel blocker for prophylaxis, diltiazem, was only selected by 44% of the countries.

##### Acute coronary syndrome

For the treatment of acute coronary syndrome a nitrate, a heparin-like medicine, a platelet inhibitor and a selective beta blocker were selected on all NEMLs while dual antiplatelet therapy (acetylsalicylic acid and an ADP-receptor blocker) was found on 41% of the NEMLs. A thrombolytic agent (e.g. rt-PA or streptokinase) which is necessary when a percutaneous coronary intervention (PCI) is not possible was on 76% of the NEMLs. From heparin-like medicines, all the countries selected unfractionated heparin (UFH) but only 15 countries (44%) additionally included low molecular weight heparins (LMWH) like enoxaparin and dalteparin. For the treatment of the most important complications of the acute coronary syndrome dopamine (recommended for cardiogenic shock) was selected in 32 countries (94%) and lidocaine (recommended for ventricular fibrillation) was selected in 26 countries (74%).

##### Heart failure

Medicines necessary to treat heart failure were on most NEMLs (94%, Fig. 2b). A loop diuretic (e.g. furosemide or bumetanide), a RAAS inhibitor, a selective beta-blocker, an aldosteron antagonist (e.g. spironolacton) and a digitalis glycoside (digoxin or digitoxin) were selected on all the NEMLs. Antihypotensives like dopamine, dobutamine or milrinone were selected on 32 (94%) of the NEMLs. At least one of the three beta-blockers for which efficacy has been shown in trials for heart failure (i.e. carvedilol, bisoprolol or metoprolol [24, 27, 48]) was included in 20 countries.

##### Atrial fibrillation

Twenty-six countries (76%) were able to cover all recommended treatment options for atrial fibrillation in their NEMLs. While medicines for frequency control (including selective beta-blockers, non-dihydropyridine calcium channel blockers and digitalis glycosides) were selected in 91% of the countries, medicines for rhythm control including pharmacological cardioversion and maintaining sinus rhythm (for medicines see Table 1) were selected in 26 countries (76%). Antithrombotic therapy with a vitamin K antagonist such as warfarin was available in 32 (94%) countries whereas direct oral anticoagulants (DOAC, e.g. dabigatran, rivaroxaban) were not included in any NEML.

##### Peripheral arterial disease

Cilostazol or naftidrofuryl are recommended for relief of ischemic complaints, which were only selected by 1 country (3%) in the current study.

##### Acute limb ischemia

Surgery is the primary intervention. After revascularization, antithrombotic therapy with platelet inhibitors is indicated which were selected by 31 of the countries (91%). However, for those patients for whom surgery is unsuitable heparin (selected in all the countries) and thrombolytic agents (selected in 76% of the NEMLs) are recommended.

## Discussion

CVDs own a substantial share in the total burden of non-communicable diseases [49]. Therefore it is important to evaluate whether adequate steps have been made by LMICs to confront this still growing burden. Selection of appropriate essential medicines on NEMLs is one of the first steps studied here in detail.

The main medicine classes for the management of CVDs were represented on NEMLs. Suboptimal selection of medicines for the prevention and treatment of CVDs was however observed for ADP receptor inhibitors, selected by less than half of the countries studied, and statins, thrombolytic agents, medicines for pharmacological cardioversion and medicines for maintaining sinus rhythm which were not selected by nearly a quarter of the countries. The total number of selected essential medicines for the prevention and treatment of cardiovascular diseases differed across income levels and regions. Over 75% of the NEMLs included adequate treatment for the primary and secondary prevention of CVDs and for the majority of acute cardiovascular events. However, management of acute coronary syndrome, especially myocardial revascularization with PCI with dual platelet therapy, could only be covered in less than half of the countries by the current selection, whereas cilostazol or naftidrofuryl for peripheral arterial disease was only found on the NEML in 1 country.

In LMICs, high cholesterol is the second highest risk factor which contributes substantially to the burden of CVDs [50]. Despite this fact, statins were missed on the NEMLs of approximately 25% of LMICs in this study while no alternative lipid lowering agents were selected in those countries. This was particularly notable in the African region. In addition, the African region had the lowest median number of essential medicines for the management of CVDs. Unlike all the other regions in the world where CVDs are the leading cause of death, in Africa mortality due to infectious diseases exceeds mortality due to CVDs [51]. Nevertheless, in the absolute term, the burden of NCDs (including CVDs) in Africa is also high and is projected to exceed communicable disease as the most common cause of death by 2030.

RAAS inhibitors were selected by all the studied countries due to extensive selection of ACE inhibitors. Nevertheless, a fraction of patients (5–35%) may not be able to benefit from ACE inhibitors because of dry cough as an adverse effect [52]. Guidelines have consensually recommended ARBs instead of ACE inhibitors for this group of patients. However, ARBs were only selected by nearly half of the countries studied.

Among non-dihydropyridine calcium channel blockers, diltiazem is the preferred treatment for stable angina pectoris, particularly in case of monotherapy [53]. Diltiazem was only selected by 44% of the countries, and is not included in the WHO model list of essential medicines [54]. Instead, the model list selected verapamil, which was also predominantly observed for the NEMLs studied. However, diltiazem is mostly well tolerated and very effective in the prophylaxis of angina whereas verapamil has negative inotropic effects and is less appropriate for this indication [23, 45].

Streptokinase was included in about 75% of the countries studied, unlike rt-PAs selected in 15% of the countries. Streptokinase, which is currently included in the complementary list of the WHO model list of essential medicines, is deemed unacceptable by some guidelines, owing to its high rate of bleeding and frequent allergic reactions (43). In addition, rt-PAs have shown to be cost-effective in both developed and developing countries [55–58]. However, infrastructural and economic constraints might have restricted their selection [59]. Similarly, LMWHs (e.g. enoxaparin) have recently been added to the WHO model list of essential medicines while they were underrepresented in a majority of the NEMLs studied. LMWHs are documented to have a better safety profile, more predictable pharmacokinetics and comparable clinical outcomes with UFH [54, 60–62].

Despite the fact that all the studied countries selected at least a selective beta blocker, the choice was dominated by atenolol (selected in all countries). Atenolol has been replaced with bisoprolol (or alternatively, metoprolol or carvedilol) for angina, arrhythmia and heart failure in the WHO model list of essential medicines [54]. As these specific beta blockers are evidence based treatments for heart failure, it is worthwhile considering them while prioritizing choices for an NEML. In the current study, these evidence based beta blockers were only found in slightly over half of the NEMLs, which varied greatly across different income levels.

Antiarrhythmic medicines indicated for rhythm control in atrial and ventricular fibrillation were absent in a quarter of countries studied while this group of medicines are life saving for patients in critical conditions. Vitamin K antagonists, e.g. warfarin were available in 32 (94%) of NEMLs whereas DOACs were not included in any of the NEMLs studied. These newer anticoagulant medicines are shown to be cost effective in health care settings in developed countries compared to vitamin K antagonists, e.g. in secondary prevention of stroke in atrial fibrillation [63, 64] albeit with a high budget impact [65]. As innovative medicines, acquisition costs of these medicines might momentarily be unaffordable for health care settings in LMICs. However, management of warfarin therapy including INR monitoring is reported to be poor in LMICs, resulting in higher rates of stroke in AF patients (between 2 and 5 time as high in LMICs compared to the developed countries) and more frequent episodes of major bleedings [66]. DOACs are shown to be superior to warfarin with respect to the occurrence of major bleeding events and do not require frequent monitoring of the anticoagulant effects [66]. This might provide additional advantages for DOACs across LMICs compared to developed countries from a health systems perspective. Yet, further studies are required to assess costs and cost effectiveness of these new medicines across LMICs [66, 67].

Essential medicines are shown to be more available compared to other medicines worldwide [7]. This indicates the importance of essential medicines to ensure access to pharmaceutical treatment. Although cardiovascular medicines were widely selected according to our analysis, selection might not have been translated in adequate access to essential cardiovascular medicines. The availability of medicines for chronic disease (including CVDs) was reported to be less than 30% in public facilities across six LMICs, while a wide variation was observed in affordability of a month of coronary heart disease treatment, ranging from 1.5 to 18.4 days of a minimum official salary [68]. Other components of access to medicines framework (particularly sustainable financing and a well-structured health care system) should as well be studied to unravel the existing suboptimal access. Integration of cost-effective non-communicable disease interventions into the basic primary health care package and finance them through sustainable financing mechanisms is necessary to support the selection of essential medicines, as recommended in the draft of the WHO NCD action plan “appendix 3” [69].

This study has a number of limitations. The number of low income countries was lower compared to the other income levels. Regional results should be interpreted cautiously since the African region was only represented by low income countries. Besides, treatment of comorbidities (e.g. diabetes) and special patient groups (e.g. elderlies, comorbid patients) were out of scope of this study. Guidelines vary in recommendations while we only included the essence of the treatments in this study, and in particular we excluded innovative medicines suggested by some of the guidelines (GP IIb/IIIa inhibitors) because of the scope of this study being limited to LMICs. Selection of oxygen and analgesics (e.g. morphine) was not assessed in the current study, but there are concerns about their availability and accessibility [70, 71]. More generally, pharmacological treatment of CVDs was the only focus of this study whereas guidelines for treatment of CVDs also incorporate non-pharmacological prevention, early diagnosis and surgical interventions. Therefore, other studies are needed to assess the integrated care options for CVDs. From a health system perspective, the example of stroke suggests that well equipped facilities are not adequately found in the public sector in LMICs, except in some upper middle income countries [59]. Geographic access to the existing centers is also a concern, where in some LMICs less than 15% of patients with stroke could reach a hospital within the first 3 h of the event [72]. It is unclear whether the observed suboptimal selection in this study, owes to differences among national treatment guidelines or not. These guidelines might exist in local languages or just be published at a national level. Nevertheless, it is questionable, whether a national guideline would not have referred to statins or thrombolytic agents. Considering the differences between health systems in countries, each jurisdiction has its specific challenges in access to medicines. This study (in part) utilises a collective approach in categorising countries (e.g. income levels), which has an inherent limitation of overlooking within category differences. There are undeniable differences between countries within each category in their economies, health systems, extent of evidence-based selection of essential medicines and use of essential medicines lists, which makes intra-category comparisons and case studies at a country level an important addition to the current study. Lastly, the latest available NEML for each country in the WHO database of essential medicine lists at the time of collection was considered to be the last update of an NEML in practice. Beyond this credible database, we were unable to verify if a country has a newer NEML unless the it appeared in the database. In studying determinants of selection, the factors we could study do not represent all potential influential factors. A plethora of issues might be involved in decision making for selection of essential medicines, including but not limited to the selection procedure itself. The results should therefore be interpreted cautiously. However, this study at least provides some insight in a number of factors which might influence the selection of medicine.

In conclusion, essential medicines for the management of CVDs were widely selected in LMICs. This has been translated into inclusion in NEMLs of essential pharmacological treatment according to evidence-based guidelines for the majority of CVDs. Nevertheless, empirical evidence suggests limited access to medicines for CVDs to this end.

## Additional file

Additional file 1: Overview of the countries included in the study**.** Including country names, World Bank income level, WHO regions, Year of (latest update) Essential Medicines List. (PDF 228 kb)

## Abbreviations

ADP: Adenosine diphosphate
AP: Angina pectoris
ARBs: Angiotensin receptor blockers
CCBs: Calcium channel blockers
CVA: Cerebrovascular accidents
CVDs: Cardiovascular diseases
LMICs: Low and middle income countries
LMWH: Low molecular weight heparins (LMWH)
NEMLs: National essential medicines lists
PCI: Percutaneous coronary intervention
RAAS inhibitors: Renin-angiotensin-aldosterone system inhibitors
rt-PA: Recombinant tissue plasminogen activator
STEMI: ST segment elevation myocardial infarction
TIA: Transient ischemic attacks
UFH: Unfractionated heparin
YLD: Years lived with disabilities
YLL: Years of life lost
