# Supplementary material for: Selection of essential medicines for the prevention and treatment of cardiovascular diseases in low and middle income countries
Source: BMC Cardiovasc Disord. 2018 Jun 25;18:126. doi: 10.1186/s12872-018-0858-5 (PMC6019804; doi:10.1186/s12872-018-0858-5)
Supplement: Supplementary file 1 — Overview of the countries included in the study. Including country names, World Bank income level, WHO regions, Year of (latest update) Essential Medicines List. (PDF 228 kb) [file 12872_2018_858_MOESM1_ESM.pdf]

**Annex 1 - Overview of the countries included in the study**

| Country | WB income level | WHO region | Year of (latest update) Essential Medicines List |
|---|---|---|---|
| **Afghanistan** | low income | Eastern Mediterranean | 2007 |
| **Argentina** | upper middle income | America | 2005 |
| **Armenia** | lower middle income | Europe | 2010 |
| **Cameroon** | lower middle income | Africa | 2009 |
| **Central African Republic** | low income | Africa | 2009 |
| **Chad** | low income | Africa | 2007 |
| **China** | upper middle income | Western Pacific | 2009 |
| **Dominican Republic** | upper middle income | America | 2005 |
| **Ecuador** | upper middle income | America | 2009 |
| **El Salvador** | lower middle income | America | 2011 |
| **Eritrea** | low income | Africa | 2010 |
| **India** | lower middle income | South-East Asia | 2011 |
| **Jamaica** | upper middle income | America | 2008 |
| **Jordan** | upper middle income | Eastern Mediterranean | 2009 |
| **Kiribati** | lower middle income | Western Pacific | 2009 |
| **Malaysia** | upper middle income | Western Pacific | 2008 |
| **Maldives** | upper middle income | South-East Asia | 2009 |
| **Mali** | low income | Africa | 2008 |
| **Morocco** | lower middle income | Eastern Mediterranean | 2008 |
| **Namibia** | upper middle income | Africa | 2008 |
| **Nicaragua** | lower middle income | America | 2009 |
| **Pakistan** | lower middle income | Eastern Mediterranean | 2007 |
| **Palau** | upper middle income | Western Pacific | 2006 |
| **Peru** | upper middle income | America | 2010 |
| **Seychelles** | upper middle income | Africa | 2010 |
| **Solomon Islands** | lower middle income | Western Pacific | 2010 |
| **Sri Lanka** | lower middle income | South-East Asia | 2009 |
| **Sudan** | lower middle income | Africa | 2007 |
| **Suriname** | upper middle income | America | 2014 |
| **Thailand** | upper middle income | South-East Asia | 2008 |
| **Tonga** | upper middle income | Western Pacific | 2007 |
| **Tuvalu** | upper middle income | Western Pacific | 2008 |
| **Uganda** | low income | Africa | 2007 |
| **Uruguay** | upper middle income | America | 2011 |
